# Introducing rapid diagnostic tests for malaria to drug shops in Uganda: a cluster-randomized controlled trial

**DOI:** 10.2471/BLT.14.142489

**Published:** 2015-01-20

**Authors:** Jessica Cohen, Günther Fink, Kathleen Maloney, Katrina Berg, Matthew Jordan, Theodore Svoronos, Flavia Aber, William Dickens

**Affiliations:** a Department of Global Health and Population, Harvard TH Chan School of Public Health, 677 Huntington Avenue, (Building 1, Room 1209), Boston, MA 02115, United States of America (USA).; b Malaria Control Team, Clinton Health Access Initiative, Boston, USA.; c Department of Health, Behaviour and Society, Johns Hopkins School of Public Health, Baltimore, USA.; d Department of Psychology, Yale University, New Haven, USA.; e Health Policy Program, Harvard University, Cambridge, USA.; f Malaria Consortium, Kampala, Uganda.; g Department of Economics, Northeastern University, Boston, USA.

## Abstract

**Objective:**

To evaluate the impact – on diagnosis and treatment of malaria – of introducing rapid diagnostic tests to drug shops in eastern Uganda.

**Methods:**

Overall, 2193 households in 79 study villages with at least one licensed drug shop were enrolled and monitored for 12 months. After 3 months of monitoring, drug shop vendors in 67 villages randomly selected for the intervention were offered training in the use of malaria rapid diagnostic tests and – if trained – offered access to such tests at a subsidized price. The remaining 12 study villages served as controls. A difference-in-differences regression model was used to estimate the impact of the intervention.

**Findings:**

Vendors from 92 drug shops successfully completed training and 50 actively stocked and performed the rapid tests. Over 9 months, trained vendors did an average of 146 tests per shop. Households reported 22 697 episodes of febrile illness. The availability of rapid tests at local drug shops significantly increased the probability of any febrile illness being tested for malaria by 23.15% (*P* = 0.015) and being treated with an antimalarial drug by 8.84% (*P* = 0.056). The probability that artemisinin combination therapy was bought increased by a statistically insignificant 5.48% (*P* = 0.574).

**Conclusion:**

In our study area, testing for malaria was increased by training drug shop vendors in the use of rapid tests and providing them access to such tests at a subsidized price. Additional interventions may be needed to achieve a higher coverage of testing and a higher rate of appropriate responses to test results.

## Résumé

**Objectif:**

Évaluer l'impact (sur le diagnostic et le traitement du paludisme) de l'introduction de tests diagnostiques rapides dans les pharmacies dans l'est de l'Ouganda.

**Méthodes:**

Au total, 2 193 ménages vivant dans 79 villages de l'étude disposant d'au moins 1 pharmacie homologuée ont été recrutés et suivis pendant 12 mois. Après 3 mois de suivi, les pharmaciens de 67 villages, choisis aléatoirement pour l'intervention, se sont vus proposer une formation leur permettant d'utiliser des tests diagnostiques rapides du paludisme et, une fois formés, un accès à ces tests à un prix subventionné. Les 12 villages restants de l'étude ont servi de villages témoins. Un modèle de régression des doubles différences a été utilisé pour estimer l'impact de l'intervention.

**Résultats:**

Les pharmaciens de 92 pharmacies ont terminé leur formation avec succès et 50 d'entre eux ont stocké et réalisé activement les tests rapides. En 9 mois, les pharmaciens formés ont réalisé une moyenne de 146 tests par officine. Les ménages ont signalé 22 697 épisodes de maladie fébrile. La disponibilité des tests rapides dans les pharmacies locales a augmenté significativement la probabilité qu'une maladie fébrile soit testée pour le paludisme de 23,15% (*P* = 0,015) et traitée par un médicament antipaludéen de 8,84% (*P* = 0,056). La probabilité que la polythérapie à base d'artémisinine soit achetée, a augmenté, de manière statistiquement non significative, de 5,48% (*P* = 0,574).

**Conclusion:**

Dans la zone de notre étude, le dépistage du paludisme a été augmenté en formant les pharmaciens à l'utilisation de tests rapides et en leur fournissant l’accès à ces tests à un prix subventionné. Des interventions supplémentaires peuvent être nécessaires pour atteindre une couverture plus élevée du dépistage et un taux plus élevé de réponses appropriées aux résultats de tests.

## Resumen

**Objetivo:**

Evaluar la repercusión en el diagnóstico y el tratamiento de la malaria de la introducción de pruebas de diagnóstico rápido en farmacias del este de Uganda.

**Métodos:**

En general, se inscribieron y vigilaron durante 12 meses 2193 hogares de 79 aldeas de estudio con al menos una farmacia autorizada. Después de tres meses de seguimiento, se ofreció capacitación en el uso de pruebas de diagnóstico rápido de la malaria a vendedores de farmacias de 67 aldeas seleccionados al azar para la intervención. Si estos ya contaban con una formación al respecto, se les ofreció acceso a este tipo de pruebas a un precio subvencionado. Las otras 12 aldeas de estudio sirvieron como controles. Se utilizó un modelo de regresión de diferencia en diferencias para estimar el efecto de la intervención.

**Resultados:**

Los vendedores de 92 farmacias completaron correctamente la capacitación y 50 proveyeron y realizaron activamente las pruebas de diagnóstico rápido. En 9 meses, los vendedores capacitados realizaron, en promedio, 146 pruebas por farmacia. Los hogares comunicaron 22 697 episodios de enfermedad febril. La disponibilidad de pruebas rápidas en farmacias locales aumentó considerablemente la probabilidad de analizar cualquier enfermedad febril en un 23,15 % para la malaria (*P* = 0,015) y de recibir tratamiento con un fármaco antipalúdico en un 8,84 % (*P* = 0,056). La probabilidad de comprar el tratamiento combinado basado en la artemisinina aumentó un 5,48 %, lo cual es estadísticamente insignificante (*P* = 0,574).

**Conclusión:**

En nuestra área de estudio, la realización de pruebas para la malaria aumentó por medio de la capacitación de los vendedores de farmacias en el uso de pruebas rápidas y la facilitación del acceso a este tipo de pruebas a un precio subvencionado. Es posible que se requieran más intervenciones para lograr una mayor cobertura de las pruebas, así como una tasa más alta de respuestas adecuadas a los resultados de las pruebas.

## ملخص

**الغرض:**

تقييم أثر تقديم الاختبارات التشخيصية السريعة إلى مخازن الأدوية في شرق أوغندا على تشخيص الملاريا وعلاجها.

**الطريقة:**

بشكل عام، تم تسجيل 2193 أسرة في 79 قرية من قرى الدراسة التي اشتملت على الأقل على مخزن أدوية مرخص وتم رصدها لمدة 12 شهراً. وبعد 3 أشهر من الرصد، تم عرض التدريب على بائعي مخازن الأدوية في 67 قرية تم اختيارها عشوائياً للتدخل وذلك في مجال استخدام الاختبارات التشخيصية السريعة للملاريا وعُرض عليهم - في حالة تلقيهم التدريب - إتاحة هذه الاختبارات بسعر مدعم. وتم استخدام قرى الدراسة الاثنتي عشرة المتبقية كمجموعات ضابطة. وتم استخدام نموذج ارتداد الفرق في الاختلافات لتقدير أثر التدخل.

**النتائج:**

أكمل البائعون من 92 مخزناً للأدوية التدريب بنجاح وقام 50 بائعاً بتخزين الاختبارات السريعة وإجرائها. وعلى مدار 9 أشهر، أجرى البائعون المدربون 146 اختباراً في المتوسط لكل مخزن. وقامت الأسر بالتبليغ عن 22697 نوبة من الأمراض الحموية. وأدى إتاحة الاختبارات السريعة في مخازن الأدوية المحلية إلى زيادة كبيرة في احتمالية اختبار أي مرض حموي للملاريا بنسبة 23.15 % (الاحتمال = 0.015) وعلاجه بأحد الأدوية المضادة للملاريا بنسبة 8.84 % (الاحتمال = 0.056). وازدادت احتمالية شراء العلاج التوليفي بالآرتيميسينين بنسبة كبيرة إحصائياً هي 5.48 % (الاحتمال = 0.574).

**الاستنتاج:**

ازداد إجراء اختبارات الملاريا في منطقة الدراسة عن طريق تدريب بائعي مخازن الأدوية في مجال استخدام الاختبارات السريعة وإتاحة هذه الاختبارات لهم بسعر مدعم. وقد تكون هناك حاجة إلى تدخلات إضافية لتحقيق تغطية أعلى للاختبارات ومعدل أعلى للاستجابات المناسبة لنتائج الاختبارات.

## 摘要

**目的:**

评估在乌干达东部药店引入快速诊断测试对疟疾诊断和治疗的影响。

**方法:**

整体而言，在79个接受研究的村庄（至少有一家执照药店）有2193户家庭纳入调查，并接受12个月的观察。经过3个月的观察，向67个乡村中随机选择进行干预的药店供应商提供使用疟疾快速诊断测试的培训，如果已经培训过，则以补贴价格为其提供此类测试的使用。剩余的12个研究村庄作为对照。使用双重差分回归模型评估干预的影响。

**结果:**

来自92家药店的供应商成功完成培训，50家积极储备和执行快速测试。在9个多月中经过培训的供应商完成了平均每间商店146次的测试。家庭报告22697起发热性疾病。当地药店快速测试的可用性让任何发热性疾病接受疟疾检测的概率显著提高23.15%（*P* = 0.015），接受抗疟药物治疗的概率也提高了8.84%（*P* = 0.056）。青蒿素联合疗法的概率有统计上不显著的5.48%的提高（*P* = 0.574）。

**结论:**

在我们的研究区域，培训药店供应商使用快速检测以及以补贴价格为其提供此类测试的使用提高了疟疾测试的概率。可能需要额外的干预来实现测试的更高覆盖率和对测试结果更高比例的正确反应。

## Резюме

**Цель:**

Оценить влияние на диагностику и лечение малярии внедрения в аптеки восточной Уганды диагностических экспресс-тестов.

**Методы:**

В общей сложности в исследование были включены и проходили мониторинг в течение 12 месяцев 2193 домохозяйства в 79 исследуемых деревнях по крайней мере с одной лицензированной аптекой. После 3 месяцев мониторинга фармацевтам аптек в 67 деревнях, случайным образом отобранных для внедрения экспресс-тестов, было предложено обучение использованию диагностических экспресс-тестов на малярию и, при условии успешного обучения, доступ к таким тестам по субсидированной цене. Остальные 12 исследуемых деревень служили в качестве контрольных. Для оценки внедрения использовалась регрессионная модель «разность разностей».

**Результаты:**

Фармацевты из 92 аптек успешно прошли обучение и 50 активно формировали запасы и выполняли экспресс-тесты. За девятимесячный период на одну аптеку приходилось в среднем 146 тестов, выполненных обученными фармацевтами. Домохозяйства сообщали о 22 697 случаях лихорадочного заболевания. Наличие экспресс-тестов в местных аптеках значительно увеличило вероятность проверки любого лихорадочного заболевания на малярию на 23,15% (*P* = 0,015) и его лечения противомалярийными препаратами на 8,84% (*P* = 0,056). Вероятность приобретения комбинированных препаратов на основе артемизинина увеличилась на статистически незначительный показатель 5,48% (*P* = 0,574).

**Вывод:**

В нашей области исследования частота тестирования на малярию была увеличена путем обучения фармацевтов аптек использованию экспресс-тестов и предоставления им доступа к подобным тестам по субсидированной цене. Для достижения более ширового охвата тестированием и более высокого процента принятия соответствующих мер по результатам тестов могут потребоваться дополнительные мероприятия.****

## Introduction

In areas where malaria is endemic, the appropriate management of febrile illness and the effective use of resources for malaria control rely on the availability and use of diagnostic tests.^1^ In the absence of diagnostic tests, antimalarial drugs are often taken for illnesses that have similar symptoms to those of malaria.^2^^–^^5^ Failure to diagnose malaria can lead to poor case management, a waste of scarce health resources and increased risk of antimalarial resistance.^1^^,^^6^ The non-treatment or delayed treatment of malaria contribute substantially to malaria-attributable child mortality.^7^^,^^8^ In Uganda, only a minority of febrile illnesses are treated with artemisinin combination therapy – i.e. the recommended first-line treatment for malaria – and many of such episodes go untreated.^9^ Similar observations have been made in Kenya, the United Republic of Tanzania and other African countries.^10^^–^^13^

The World Health Organization (WHO) recommends parasitological confirmation of malaria before antimalarial drug use.^14^ Although the current *Global malaria action plan* of the Roll Back Malaria Initiative calls for universal access to malaria testing,^15^ such access remains a distant goal in most countries with endemic malaria. A study in six African countries found that only 4–31% of children with febrile illnesses were tested for malaria.^9^ In many countries, patients and caregivers rely heavily on a loosely regulated private sector for malaria treatment.^16^^,^^17^ In consequence, the engagement of the private sector has become an increasingly common strategy in malaria control programmes – as reflected, for example, in the pilot Affordable Medicines Facility–malaria (AMFm).^18^

The development of inexpensive and simple rapid diagnostic tests for malaria has opened the possibility of widespread access to malaria diagnosis. These antigen detection tests have been shown to be as effective as routine microscopy in malaria diagnosis^19^ and can be safely performed by individuals with only basic training.^20^ Although research from Cambodia,^21^ Somalia^22^ and Uganda^23^ has shown that the distribution of rapid diagnostic tests by the private sector is feasible, we know very little of the impact of this approach on population-level rates of malaria diagnosis and purchase of antimalarial drugs. We therefore conducted a trial in eastern Uganda to investigate the impact – on malaria diagnosis and the purchase of antimalarial drugs – of training the vendors from licensed drug shops to test patients with a rapid diagnostic test for malaria. The trained vendors were also encouraged to buy the test, at a subsidized price, from local wholesale providers. The study took place in Uganda’s eastern region, where the annual transmission rates for malaria exceed 100 infective bites per person^24^ and presumptive symptom-based treatment remains common – especially when, as commonly occurs, treatment is sought outside the higher level public-health facilities.^25^^–^^27^ Malaria is responsible for 30–50% of outpatient visits and 9–14% of inpatient deaths in Uganda.^28^

## Methods

The study was designed as a cluster-randomized controlled trial, with extensive monitoring of the health behaviour of households before and after a rapid diagnostic test was made available in local drug shops. Since the study was designed to explore both cross-sectional and pre-post differences, 67 (85%) of the study villages were randomly selected to receive the intervention while the remaining 12 (15%) were used as a control group. The study was implemented between March 2011 and April 2012. Training in the use of the diagnostic test occurred between 21 June and 6 July, 2011. A simple random number draw – generated by Stata/SE version 11.0 (StataCorp. LP, College Station, United States of America) – was used for the selection of study villages and households and the assignment of villages to the intervention or control arm.

### Villages

**Fig. 1:**
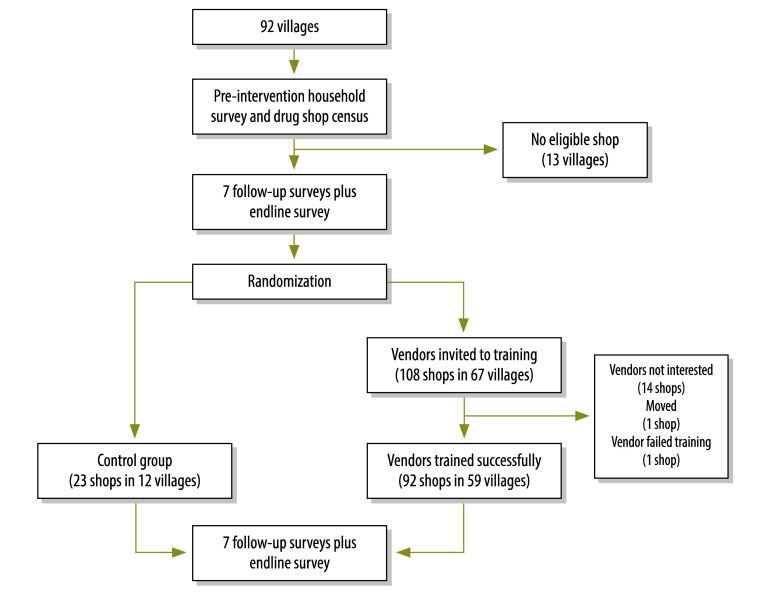
Study design: introduction and use of rapid diagnostic tests for malaria in drug shops, Uganda 2011–2012

The sampling frame for the study included all of the villages in the districts of Budaka, Bukedea, Kibuku, Kumi, Ngora and Pallisa that had at least one drug shop licensed and registered with the ministry of health. Although the district health office initially identified 92 such villages, only 79 still had at least one licensed drug shop at the time the main study was launched. These 79 villages were included in the cluster randomization (Fig. 1).

### Households

The households in all 92 target villages were listed before the launch of the study. Subsequently, 25 households in each target village were randomly selected, visited for a baseline survey to record basic demographic characteristics and health behaviours, and re-visited every month for 9 months (Fig. 1) to monitor their health problems and treatment seeking. During the tenth and final follow-up survey – i.e. the so-called endline survey – all consenting adults and – if their caregivers gave consent – the children in the study households were tested for malaria using the rapid diagnostic test.

### Drug shops

Each licensed drug shop listed by the ministry of health in the 79 villages and included in the randomization was visited for a baseline survey in April 2011 and an endline survey in March 2012.

### Intervention

Vendors from 108 drug shops in the 67 intervention villages were invited for 2 days of training in the use of a rapid diagnostic test for malaria – i.e. the CareStart Malaria HRP2 (Pf) test (Access Bio, Somerset, USA). The training, described previously,^23^ was facilitated by trainers approved by the national ministry of health. These trainers reviewed the signs and symptoms of uncomplicated malaria and severe illness and instructed the trainees on how to perform the diagnostic test and on the recommended first-line treatment for malaria. The first day of training was classroom-based while the second day involved practical experience in a health facility. Trainees were not given specific instructions on when to recommend testing and – apart from the promotion of artemisinin combination therapy – were not given treatment algorithms. No retail price for the test was suggested. Our main objective was to observe whether and how the trainees chose to integrate the test into their normal practice. Upon successful completion of the training, each trainee was given a free box of 40 test kits, gloves, an instruction leaflet, results slips and a sharps disposal box. Any new staff employed by the drug shops in the intervention villages after the initial training were invited to a similar training course that was run in October 2011.

Trained drug shop vendors were visited monthly to track their stocking and usage of the rapid diagnostic test and compliance with the recommended protocols for testing. Test kit storage, administration and post-use disposal were monitored using a 17-point checklist. Information on the shop’s stock of the test kits and price of the test for patients was recorded. At the same time, any questions the vendors had about the test’s administration were answered. Every 3 months, four unused test kits were collected from each shop holding the kits and sent for lot testing at the Foundation for Innovative Diagnostics Laboratory at the Pasteur Institute of Cambodia, in Phnom Penh. Every kit investigated in this way passed lot testing.

### Test kits

According to WHO, the test investigated has a panel detection score of 98.7%, a false-negative rate of < 1% and a total false-positive rate of 2.4%.^29^ Because this test, like other tests based on the detection of histidine-rich protein II, can remain positive for up to 5 weeks after a cured infection, a higher false-positive rate may occur in settings where malaria is highly endemic.^30^ Trainees were told that any patient who tested positive who had also taken antimalarial drugs in the previous 4 weeks should be referred to a facility where they could be checked for malarial infection by microscopy.

The test kits were purchased and imported, at a cost of 0.70 United States dollar (US$) per kit, by the study team. They were then sold to a prominent pharmaceutical wholesaler in Kampala at a subsidized price of US$ 0.12 per kit. The wholesaler’s regional pharmacy in the city of Mbale subsequently sold the kits – exclusively to our trainees – at a price of US$ 0.19 per kit.^23^

### Data entry and analysis

Data were entered using the CSPro 4.0 package (United States Census Bureau, Suitland, USA) and mainly analysed, using Stata version 11.0, in a multivariate linear probability difference-in-differences model. A *P*-value of 0.05 or less was considered statistically significant. The primary outcome was whether a household member with febrile illness was tested for malaria. Secondary outcomes included the medication taken to treat febrile illness – if any – and where treatment – if any – was sought. The main independent variable was whether the illness occurred in a village in our intervention or control arm. As vendors from 15% of the drug shops targeted for training did not complete their training, intention-to-treat effects were estimated.

To control for seasonal effects and for village characteristics, a full set of village and monthly fixed effects were included in the fully adjusted model. Estimated robust standard errors were clustered at the village level.^31^^,^^32^ Although adjustments were also made to remove the potential effects of a behaviour change campaign that was rolled out in the later stages of our intervention, the roll-out of the campaign was orthogonal to the main treatment and did not affect our main results.

Our study was powered to detect an increase in the fraction of fever cases seeking health care at drug shops using the rapid diagnostic kit from 10% to 20%. Assuming an incidence of one episode of febrile illness per person-year, a mean household size of five individuals, 40% of households seeking treatment at a drug shop and an intra-class correlation of 0.05, the study was powered to detect the targeted 10% difference in the 9-month intervention period with a probability of 0.92.

### Ethics

Ethical approval for this study was given by the Harvard School of Public Health (protocol # P19371–106) and the Uganda National Council for Science and Technology (protocol # HS805).

### Trial registry

The trial was registered as clinical trial NCT01652365 at clinicaltrials.gov.

## Results

### Drug shops

Out of the 108 registered drug shops, 92 shops completed training. Fourteen vendors declined to be trained, one shop was relocated outside our study area and the vendor from another shop was considered to have failed the training. Each of the 92 shops had at least one staff member who successfully completed training (Fig. 1).

Over the monitoring period from July 2011 to March 2012, shops run by successful trainees bought a mean of 146 (median: 40) diagnostic kits from the local wholesaler (Table 1). Overall, 13 440 test kits were bought and the shops investigated 10 412 patients using the test kits that they had been given or bought. However, 37 such shops (40%) did not purchase any of the test kits and most of the others bought only small numbers of the kits (Table 2). Together, just three shops accounted for 32% (3346) of all of the rapid diagnostic tests performed on patients.

**Table 1:** Drug shop purchases and use of rapid diagnostic tests for malaria, Uganda, July 2011–March 2012

Rapid diagnostic test	Mean	Median	SD	Min	Max
No. purchased	146.09	40.00	261.71	0	1320
No. performed	113.17	10.00	234.92	0	1220
Retail price (Ugandan shillings)^a,b^	1125.00	1000.00	293.52	500	2000

Max: maximum; Min: minimum; SD: standard deviation.

^a^ At the time of the study, 10 000 Ugandan shillings were equivalent to 3.82 United States dollars.

^b^ Values are for 87 of the 92 shops.

**Table 2:** Frequency distributions for drug shop purchases and use of rapid diagnostic tests for malaria, Uganda, July 2011–March 2012

No. of tests/shop	Purchase of tests			Performance of tests	
	No. of shops	% of purchases		No. of shops	% of tests performed
0	37	0.0		42	0.0
1–100	24	10.1		26	8.4
101–500	24	41.5		18	41.7
501–1000	4	21.9		3	17.8
> 1000	3	26.5		3	32.1

The mean price paid by a patient assessed with the rapid diagnostic kit was US$ 0.43 – representing a 125% markup on the kit’s wholesale price. According to the data collected in our household surveys, the median prices paid for artemisinin combination therapy for individuals younger and older than 5 years were US$ 0.57 and US$ 0.76, respectively. The corresponding costs of quinine – which accounted for 72% of purchases of antimalarial drugs other than artemisinin combination therapies – were US$ 0.57 and US$ 0.38, respectively.^12^

### Households

**Fig. 2:**
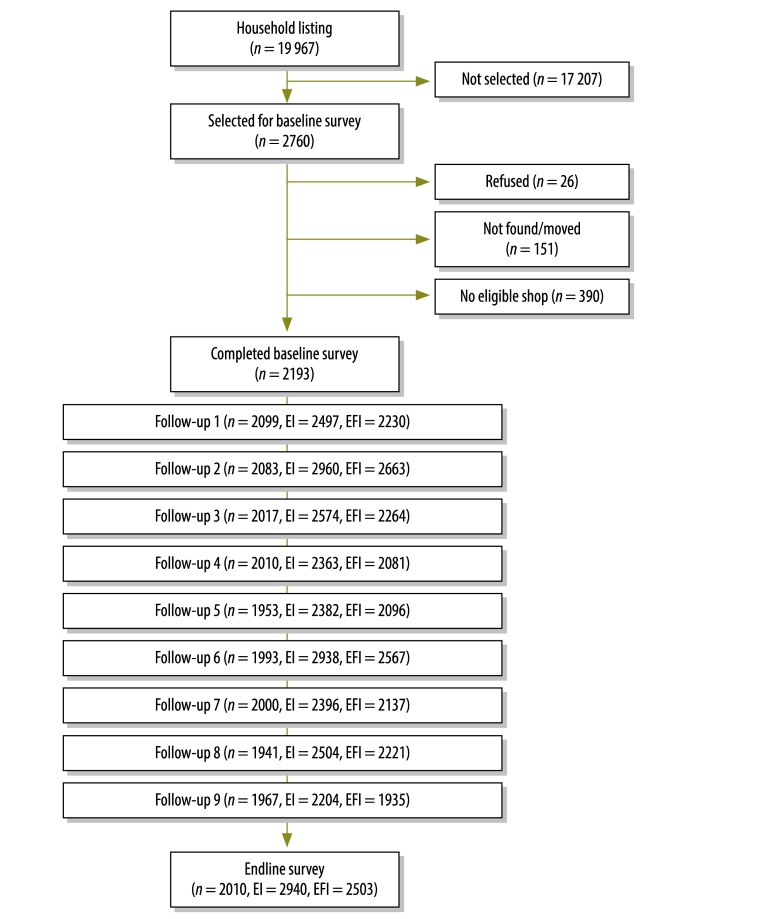
Monitored households and observed morbidity, Uganda, March 2011–April 2012

EI: episodes of illness; EFI: episodes of febrile illness.

Fig. 2 illustrates the households investigated and illnesses captured over the study period. In total, 25 758 episodes of illness were reported by study households across the 10 survey rounds. Respondents reported the presence of fever in 22 697 (88.1%) of these episodes and we focused on these episodes of febrile illness in our analysis. For 4364 of the reported episodes of illness – including 3908 episodes of febrile illness – treatment was sought at one of the study drug shops.

The study households in the intervention and control villages appeared similar in terms of their baseline demographic characteristics (Table 3). The estimated prevalence of malaria at the time of the endline survey was 43% in both arms of the study. Table 4 presents the characteristics of the episodes of febrile illness recorded pre-intervention, in monthly morbidity surveys. Most individuals (4448) with febrile illness sought treatment in the public sector, at a private-sector drug shop or pharmacy, or at a private-sector clinic or hospital. However, 21.5% (1218/5666) of such individuals sought no care. Roughly half (1039/2322) of the reported visits to drug shops for the treatment of febrile illness were to a shop that was involved in our study.

**Table 3:** Baseline characteristics of the surveyed households, Uganda, March–April 2011

Characteristic	Households in control villages (*n* = 326)	Households in intervention villages (*n* = 1867)	Difference, percentage point^a^	*P*^b^
**Mean no. of individuals in household (SD)**				
All ages	6.62 (3.32)	6.34 (3.36)	−0.28	0.312
Aged < 5 years	1.16 (1.07)	1.06 (1.05)	−0.01	0.193
**Fraction of household members sleeping under bednets, %**	58.7	60.6	1.9	0.579
**No. of households own land (%)**	237 (72.7)	1385 (74.2)	1.5	0.864
**No. of households treat drinking water (%)**	70 (21.5)	452 (24.2)	2.7	0.478
**No. of heads read English (%)**	112 (34.4)	564 (30.2)	−4.1	0.476
**No. of households have at least one mobile phone (%)**	207 (63.5)	1099 (58.9)	−4.6	0.396
**No. of households have at least one bicycle (%)**	182 (55.8)	1088 (58.2)	2.4	0.534
**No. of households have electricity (%)**	26 (8.0)	185 (9.9)	1.9	0.595

SD: standard deviation.

^a^ For mean number of individuals, the values represent numbers.

^b^ Adjusted for clustering at village level.

**Table 4:** Pre-intervention treatment-seeking for febrile illness, Uganda, April–June 2011

Characteristic	No. of episodes of febrile illness in households (%)		Difference, percentage point	*P*^a^
	In control villages (*n* = 805)	In intervention villages (*n* = 4861)		
**Episode in household member aged < 5 years**	319 (39.6)	1585 (32.6)	−7.0	0.019
**Episode in female household member**	436 (54.2)	2734 (56.2)	2.1	0.257
**Treatment seeking by affected household member**				
Visited public hospital or clinic	322 (40.0)	1446 (29.7)	−10.3	0.073
Visited private hospital or clinic	180 (22.4)	1105 (22.7)	0.4	0.953
Visited any drug shop or pharmacy	305 (37.9)	2017 (41.5)	3.6	0.592
Visited study drug shop	105 (13.0)	934 (19.2)	6.2	0.126
Sought any care	669 (83.1)	3779 (77.7)	−5.4	0.126
**Malaria testing of affected household member**				
Received malaria test	306 (38.0)	1258 (25.9)	−12.2	0.002
And visited public hospital or clinic^b^	188/300 (62.7)	698/1219 (57.3)	−5.4	0.398
And visited private hospital or clinic^b^	54/135 (40.0)	235/700 (33.6)	−6.4	0.565
And visited any drug shop or pharmacy^b^	16/198 (8.1)	89/1518 (5.9)	−2.2	0.444
And visited study drug shop^b^	10/105 (9.5)	75/934 (8.0)	−1.5	0.689
**Medication taken by affected household member**				
Artemisinin combination therapy	289 (35.9)^c^	1419 (29.2)^d^	−6.7	0.092
Any antimalarial drug	549 (68.2)	2641 (54.3)	−13.9	0.003
Any antibiotic	236 (29.3)	1258 (25.9)	−3.4	0.227

^a^ Adjusted for clustering at village level.

^b^ Denominator represents the number of people that visited the facility.

^c^ Representing 52.6% of those who took any antimalarial drug.

^d^ Representing 53.7% of those who took any antimalarial drug.

During the pre-intervention period, malaria testing and antimalarial drug use among cases of febrile illness were significantly less likely in the intervention villages than in the control villages – 25.9% (1258/4861) versus 38% (306/805; *P* = 0.002) and 54.3% (2641/4861) versus 68.2% (549/805; *P* = 0.003), respectively. This difference appears to be a reflection of a relatively larger fraction of patients from the control villages seeking treatment at a public facility. In the pre-intervention period, the percentage of febrile patients seeking care in the private sector who were tested for malaria was similar across the two study arms (40% [54/135] versus 33.6% [235/700]; *P* = 0.565).

Table 5 shows the population-level crude estimates and the corresponding – and, generally very similar – adjusted estimates of the intervention’s impact on testing, medication choice and treatment seeking. Among all cases of febrile illness and among cases of febrile illness in children younger than 5 years, according to the crude model, the intervention significantly increased the probabilities of being tested for malaria, by 6.0 (*P* = 0.015) and 7.6 percentage points (*P* = 0.015), respectively. According to the same model, the intervention increased the likelihood of taking any antimalarial drug or artemisinin combination therapy by 4.8 and 1.6 percentage points and reduced antibiotic usage by 0.3 of a percentage point – but none of these differences reached statistical significance. Appendix A (available at: https://cdn1.sph.harvard.edu/wp-content/uploads/sites/358/2012/08/AppendixA.pdf) demonstrates the robustness of these main results, which remained similar if we (i) used patient-reported episodes of suspected malaria rather than all fever episodes, (ii) confined our analysis to the episodes of illnesses that occurred before the roll-out of the behaviour change campaign, or (iii) used logistic regression instead of linear probability models.

**Table 5:** Impact of intervention on treatment seeking, malaria testing and drug use in response to episodes of febrile illness, Uganda, July 2011–March 2012

Variable	No. of episodes included in model	Results from unadjusted model				Results from adjusted model^a^		
		*β*	95% CI	% change^b^		*β*	95% CI	% change^b^
**Malaria testing of affected household member**								
Member of any age	22 560	0.060	0.012 to 0.109	23.15		0.057	0.010 to 0.104	21.99
Member aged < 5 years	8090	0.076	0.015 to 0.138	24.57		0.066	0.008 to 0.124	21.34
**Medication taken by affected household member**								
Artemisinin combination therapy	22 697	0.016	−0.041 to 0.074	5.48		0.020	−0.037 to 0.076	6.85
Artemisinin combination therapy, among members aged < 5 years	8134	−0.009	−0.084 to 0.065	−2.80		−0.014	−0.088 to 0.061	−4.35
Artemisinin combination therapy, among members who took any antimalarial drug	12 210	−0.004	−0.076 to 0.068	−0.74		−0.006	−0.069 to 0.057	−1.12
Any antimalarial drug	22 697	0.048	−0.001 to 0.098	8.84		0.052	0.002 to 0.102	9.58
Any antibiotic	22 697	−0.003	−0.055 to 0.049	−1.16		0.007	−0.048 to 0.062	2.70
**Treatment seeking by affected household member**								
Visited public hospital or clinic	22 697	0.012	−0.036 to 0.060	4.03		0.024	−0.019 to 0.068	8.07
Visited private hospital or clinic	22 697	−0.019	−0.069 to 0.032	−8.36		−0.023	−0.075 to 0.029	−10.12
Visited any drug shop or pharmacy	22 697	0.042	−0.040 to 0.124	10.12		0.040	−0.040 to 0.119	9.64
Visited study drug shop	22 697	−0.026	−0.085 to 0.032	−16.47		−0.027	−0.090 to 0.036	−14.06
Sought any care	22 697	0.029	−0.021 to 0.078	3.73		0.034	−0.014 to 0.083	4.38

CI: confidence interval.

^a^ Adjusted for month and village fixed effects.

^b^ Percentage change in outcome: (beta/pre-intervention mean in intervention areas) x 100.

**Fig. 3:**
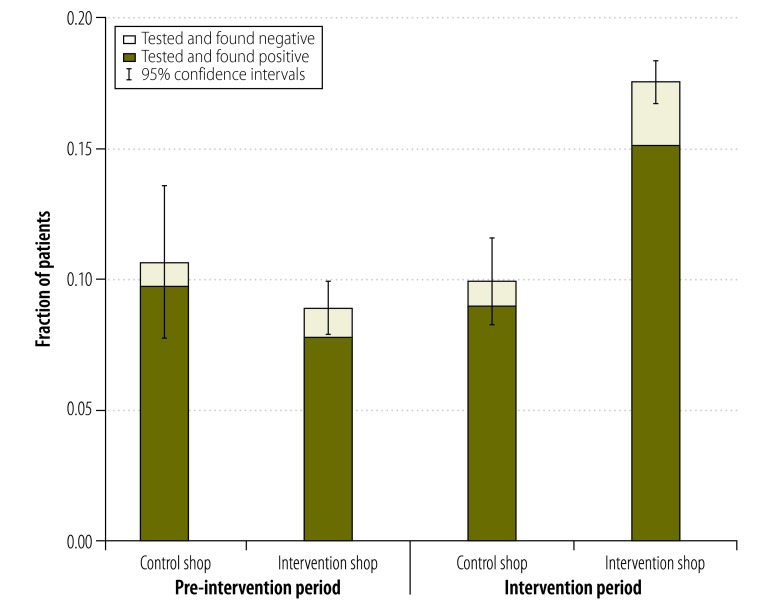
Testing rates for malaria and results for patients visiting study drug shops, Uganda, March 2011–April 2012

Fig. 3 shows the fractions of patients visiting the drug shops in intervention and control villages who were tested for malaria before and after roll-out of the intervention. Prior to the first training course about the rapid diagnostic test, the fraction of patients tested for malaria in a study drug shop was similar in the intervention and control villages, 8.9% (70/786) and 10.6% (12/113), respectively. After the first training course, the fraction of patients tested for malaria in a study drug shop in the control arm remained almost unchanged (33/334) but the corresponding value in the intervention arm almost doubled (390/3011; *P* < 0.001). Nearly 90% (3112/3458) of patients investigated using the rapid diagnostic test gave a positive result. The reliability of the test results is discussed in Appendix A.

**Fig. 4:**
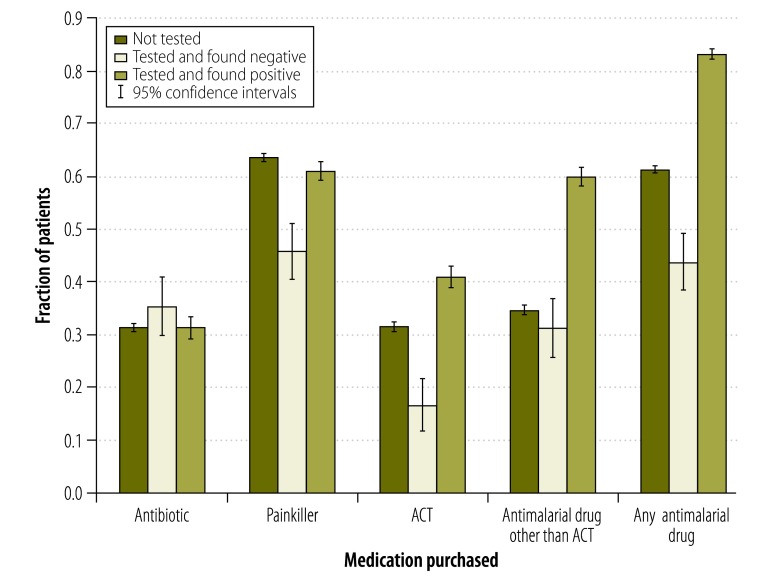
Medication taken by febrile patients visiting drug shops that had vendors trained to use the malaria rapid test, Uganda, March 2011–April 2012

Fig. 4 summarizes our data on the patients who, during the intervention period, visited and were tested at the study drug shops in the intervention villages. Although over 80% (285/342) of such patients who tested positive for malaria purchased an antimalarial drug of some kind, nearly 45% (21/48) of those who tested negative did the same. Test-positive patients were more than twice as likely to purchase artemisinin combination therapy as test-negative patients (40.9% [140/342] versus 16.7% [8/48]; *P* < 0.001). However, less than half of the test-positive patients – and 31.5% (590/1871) of the untested patients – purchased artemisinin combination therapy. We found no differences in antibiotic purchases according to testing status or test result.

ACT: artemisinin combination therapy.

Notes: Medication is shown separately for the patients who were tested for malaria with a rapid diagnostic kit and found positive, the patients who were similarly tested and found negative and the patients who were not tested.

## Discussion

In countries where malaria is highly endemic, many episodes of febrile illnesses are treated at drug shops. Our results indicate that, by offering training and access to subsidized rapid diagnostic tests to private drug shops, it is possible to increase malaria testing rates significantly. Three of the most commonly voiced concerns regarding use of rapid tests for malaria diagnosis by the private sector are poor adherence to protocols, the potential crowding out of public-sector treatment and increased antibiotic purchases by patients who have a negative result. We found no evidence to support any of these concerns. In addition, as we previously reported, our monthly monitoring of the drug shops indicated generally high levels of compliance with recommended treatment, storage, waste management and test-kit administration protocols.^23^

From a policy perspective, a major challenge for any health initiative in the private sector is the achievement of adequate uptake and coverage. In our study setting, the potential impact of the intervention was limited by the need to work only with drug shops that were licensed by the Ugandan Ministry of Health. Inclusion of unlicensed outlets would have substantially increased the reach of our programme but may also have complicated monitoring and quality control. Even among the licensed shops with trained staff, however, uptake was limited. Only about 60% (55/92) of such shops chose to stock the test kits and fewer than 20% (390/2261) of the febrile patients who visited a drug shop that had a trainee were actually tested for malaria. While no detailed information on the reasons for the relatively weak uptake of the rapid tests was collected, higher rates of uptake could probably be achieved by combining behavioural change efforts with stronger financial incentives. The price subsidies for the test kits could be increased, drug shops could be paid incentives to perform tests and provide appropriate treatment to the test-positive patients, and the level of any subsidy could be correlated with the quality of the testing provided by each shop. Greater impact could potentially also be achieved if the test-related training and subsidy could be combined with training on the appropriate treatment of non-malarial illnesses – although this would require policy-makers to support the movement of non-medical professionals further into formal case management.

A second main challenge, from a policy perspective, is the remarkably low uptake of artemisinin combination therapies that we observed. Only 40.9% of the patients who tested positive for malaria actually purchased such therapies. This modest amount of uptake does not appear to have been driven by lack of access, given that 84.2% (96/114) of our study drug shops reported having artemisinin combination therapies in stock at the time of our endline survey.^12^ It is possible that patients perceived the prices of such therapies to be too high – compared with those of other antimalarial treatments – even though, in Uganda at the time of our study, the prices of such therapies had been substantially lowered by subsidies from the Affordable Medicines Facility–malaria programme.^12^^,^^18^

Furthermore, use of rapid tests for malaria diagnosis in the private sector appears to be a feasible and potentially effective way to increase testing rates and improve overall case management. As discussed in greater detail elsewhere,^33^ the subsidizing of such tests for use in the private sector is likely to yield highest returns in settings where malaria prevalence is low and treatment seeking in the private sector is common. The accurate diagnosis of malaria could eliminate the wasteful use of antimalarial drugs for non-malarial illness and improve the management of malaria and other febrile illnesses – particularly if the private sector is properly incentivized and equipped to treat the true causes of non-malarial illness appropriately.
